# Comparison of Dermal Substitutes in Wound Healing Utilizing a Nude Mouse Model

**Published:** 2005-03-14

**Authors:** Anh-Tuan N. Truong, Areta Kowal-Vern, Barbara A. Latenser, Dorion E. Wiley, Robert J. Walter

**Affiliations:** a Metropolitan Hospital Group General Residency in Surgery Program, Chicago, IL; b Department of Trauma, Sumner L. Koch Burn Center, John H. Stroger, Jr., Hospital of Cook County, Chicago, IL; c University of Iowa Hospitals and Clinics, Iowa City

## Abstract

**Background:** Dermal skin substitutes have become a standard of care in burn treatment. **Objective:** To compare and assess wound contracture reduction and histologic incorporation into the wound, dermal substitutes were implanted into full-thickness skin wounds in nude mice. **Materials and Methods:** Thirty-seven mice received a full-thickness 2 × 2 cm dorsal skin wound, and were either implanted with an acellular dermal matrix, Alloderm, Dermagraft-TC, Dermalogen, or Integra or assigned to the control group (with no dermal substitute). At 28 days postsurgery, the wounds were assessed for contraction, epithelialization, and other histological characteristics. **Results:** Each dermal substitute decreased wound contracture, but Alloderm and the acellular dermal matrix did so significantly compared to the control (*P* < .01 and *P* < .03, respectively). Within-group and control comparisons showed no significant differences with respect to the presence of dystrophic calcification, squamous hyperplasia, infiltration of neutrophils, fibroblasts, and macrophages, epidermal keratinocyte stratification, or collagen fiber configuration. **Conclusions:** Integra elicited the greatest foreign body response. Although the Dermalogen group had the thickest elastin fiber fragments, Dermagraft may have initiated the earliest elastin fiber formation in the wounds. While all dermal substitutes were incorporated into the wound bed and wound contracture was decreased, acellular dermal matrix and Alloderm, both human skin–derived products, produced less contraction and the thickest new “dermis” in the healed wounds compared to the control or synthetic dermal substitutes.

Early excision of the burn eschar has greatly improved burn patients' survival. In some cases, the patient is left with extensive regions devoid of dermis and poor cosmesis. Currently, a variety of skin substitutes or artificial dermal replacements are used not only to decrease morbidity and wound contracture in severely burned patients but also to enhance cosmesis of partial- and full-thickness burn wounds.^1^–^9^ Dermal substitutes serve as a scaffold into which cells can migrate and repair the injury. Although dermal substitutes and their histology have been described in the literature and several have been compared to a split-thickness skin graft as the “criterion standard,” there have not been any studies comparing the efficacy of these dermal substitutes as a group in respect to wound contracture and histologic features. The purpose of this study was to compare the effectiveness of several dermal substitutes in an animal model of wound healing. Human cadaver skin was not used because it is viable rather than processed tissue. For this reason, acellular dermal matrix (ADM) was a more compatible dermal substitute for this study because it was a processed nonviable dermal substitute derived from human cadaver skin. We hypothesized that human skin–derived dermal substitutes (ADM, Alloderm, and Dermalogen) would generate a thicker dermis with less wound contraction than would control (Fibrin Sealant Tisseel VH) or synthetic matrices (eg, Dermagraft-TC, Integra). The study compared the wound healing attributes of various dermal substitutes grossly and histologically.

## MATERIALS AND METHODS

### Dermal matrices and fibrin glue

*ADM* was prepared as described below and in the literature by Takami et al.^10^ It is a dermal collagen matrix derived from banked human skin that is treated to remove all cellular components.^11^

*Alloderm* (LifeCell Corporation, Branchburg, NJ) is a dermal collagen matrix derived from banked human skin that is treated to remove most cellular components. It is freeze-dried for shipping and storage.

*Dermagraft-TC* (Advanced Tissue Sciences, La Jolla, Calif) is composed of a woven bioabsorbable polymer (polyglycolic and polylactic acids) membrane within which human dermal fibroblasts are grown and then devitalized. It is used for reepithelialization of middermal and mid-to-deep–dermal indeterminate burn wounds and contains Type I collagen fibers, glycosaminoglycans, and growth factors such as TGF-beta and decorin. This product is not used as a dermal substitute in humans, although it was used as such in this study.

*Dermalogen* (Collagenesis, Beverly, Mass) is a powdered human dermal collagen matrix that is treated to remove some cellular components, is freeze-dried, and is then milled into a fine powder. The collagen concentration was mixed with RPMI (MP Biomedicals, Inc, Aurora, Ohio) to form a 15 mg/mL solution. It is used primarily for aesthetic plastic surgery, as a “filler.”

*Integra* (Integra Life Sciences Corporation, Plainsboro, NJ) is a bilayer artificial skin replacement with a “dermal” layer composed of bovine collagen gel cross-linked with shark chondroitin-6-sulfate. The synthetic “epidermal” layer is composed of a polysiloxane polymer that was removed before use in this study.

*Fibrin Sealant Tisseel VH* (Baxter Health, Deerfield, Ill) is a 2-component fibrin glue mixture: fibrinogen + calcium and thrombin + aprotinin (protease inhibitor) were combined quickly and dispensed onto a wound, forming a fibrin clot.

### Preparation of ADM

Cryopreserved normal human skin (U.S. Tissue & Cell, Cincinnati, Ohio) obtained from cadavers, using a dermatome set at 0.012 in thick, was thawed rapidly at 37°C. It was then treated with 2.5 units/mL Dispase II (Boehringer Mannheim, Indianapolis, Ind) in phosphate-buffered saline containing 0.2 mM CaCl_2_ at 4°C for 24 hours to remove epidermis and other cellular components from the dermal matrix. Subsequently, the dermal matrix was incubated in buffered 0.5% Triton X-100 (United States Biochemical Corp, Cleveland, Ohio) for 24 hours at room temperature with continuous shaking. ADM was extensively washed with phosphate-buffered saline and stored in phosphate-buffered saline at 4°C until use. All solutions used for ADM preparation were filter-sterilized, and all procedures were performed aseptically. Sodium azide (0.02% w/v) was present at all times in the extraction solutions to prevent microbial growth, and was thoroughly washed out before implanting the ADM.

### Animals and surgery

Thirty-seven male NIH Swiss nude mice (NSWNU, homozygous, outbred, 4–5 weeks, 20–25 g) were used. The study was reviewed and approved by the Institutional Animal Care and Use Committee. Animals were housed in sterilized cages with sterile feed, water, and bedding. A single dose of antibiotic (kanamycin; 25 units/kg) was administered intramuscularly to all animals approximately 1 hour prior to surgery. All survival surgery was performed using aseptic technique in the Animal Facility operating rooms. Anesthesia consisted of ketamine/xylazine (150/8 mg/kg) administered intraperitoneally with additional ketamine (30 mg/kg) given intraperitoneally as needed to maintain deep anesthesia. Standardized full-thickness 20 mm × 20 mm skin wounds (removing the panniculus carnosus muscle layer) were excised from the dorsum of each anesthetized mouse. On Day 0, the mice weighed (mean ± SD) 27 ± 3 g and on Day 28, they weighed 31 ± 2 g, showing an overall weight gain of 4.6 ± 2 g. Mice with the Alloderm implant gained the most weight (6.5 ± 2 g) compared to the other groups. The weight gain included the weight of the implanted dermal substitute, which was not weighed at the time of implantation.

### Implantation of dermal substitutes

There were 6 study groups: ADM, Alloderm, Dermagraft-TC, Integra, Dermalogen, and control. The control group had the same type of wound and wound treatment as the other groups but did not receive a dermal substitute. Dermalogen solution (0.75 cc) was placed into the wound; skin at the wound corners was sutured to the underlying muscle. Each of the dermal matrices was cut to the size of the wound and sutured to the adjoining skin and the underlying muscle at their corners using nylon suture. The Integra silicone layer was removed after the Integra gel was placed into the wound. Fibrin Sealant Tisseel VH was sprayed on the wounds, which were then covered with semipermeable adhesive film (Op-Site, Smith and Nephew, Largo, Fla), Xeroform (Sherwood Medical, St. Louis, Mo), and dry cotton gauze. This dressed wound was then covered with fine stainless steel mesh fixed onto the animal's back with 4 sutures to the skin, each about 1 cm distant from the wound. The mesh was used to prevent the wounds from being disturbed by chewing or scratching. Animals and wounds were inspected daily to ensure that the dressings were intact. After 2 to 3 weeks postsurgery, the wounds were partially healed and the stainless steel mesh was removed. At the end of the 4-week study period, all animals were euthanized and the wounds were harvested, fixed in 10% buffered formalin, cut into five to seven 2-mm sections, and prepared for histological analysis.

### Gross and histologic measurements and assessments

Wound characteristics were measured grossly as well as histologically on Day 28 postsurgery. Direct measurements of wound area and the extent (area) of epithelialization and wound contraction were determined from digital photographs (Olympus C3030) using computer planimetry software (UTHSCSA ImageTool for Windows v. 2.00). All excised healed wounds were paraffin-embedded, and sections were stained with Hematoxylin & Eosin, Masson Trichrome, and Elastin Van Gieson. A “blinded” pathologist (A.K.V.) evaluated all samples histologically for depth and length of the healed wound area, epithelial stratification, incorporation of the dermal substitute, degree of neutrophil, macrophage, fibroblast, and foreign body giant (FBG) cell infiltration, and extent of elastin formation. This examination was performed using an Olympus microscope with a millimeter ruler eyepiece adapter. Histologic assessment was performed in low-power fields (100× magnification) on all cross sections of each wound area.

### Statistical analysis

Study groups were compared utilizing Statistica (Statsoft, Tulsa, Okla). Summary descriptive statistics such as median, means, standard deviation and error, 1-way analysis of variance, chi-square 2 × 2 summary frequencies (Pearson and Maximum Likelihood), and the Tukey test for unequal numbers were calculated. Nonparametric analyses utilized the Kruskal-Wallis analysis of variance by Ranks and Mann-Whitney *U* test. A *P* value of less than .05 was considered significant.

## RESULTS

### Wound contracture

Table 1 shows the dimensions of the healed wounds at 28 days postsurgery for each group.

**Figure 1:**
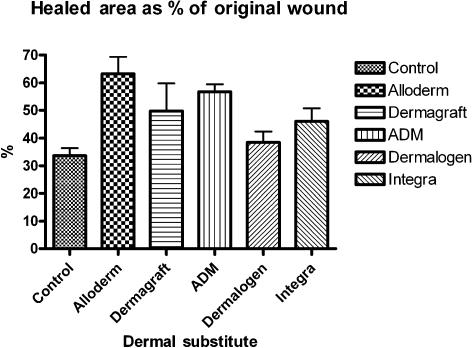
Size of the 28-day wound as a percentage of the original wound surface area. ADM indicates acellular dermal matrix.

Dermal substitute implantations and control decreased wound contraction when assessed by percentage retention of the original wound area: Control by 34% ± 7%; Dermalogen by 39% ± 9%; Integra by 46% ± 12%; Dermagraft-TC by 50% ± 22%. Based on the initial 400 mm^2^ wound surface area, Alloderm and ADM underwent the least contracture and retained the greatest mean surface area compared to control and other dermal substitutes (63% ± 14% [*P* < .01] and 57% ± 7% [*P* < .03) total wound surface area, respectively), Figure 1.

### Epithelialization

**Figure 2:**
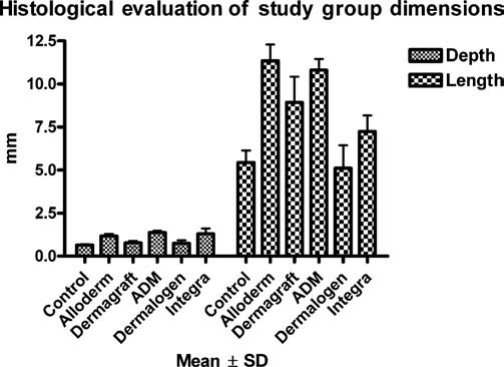
Healed wound dimensions (depth and length histologically) at 28 days postsurgery. ADM indicates acellular dermal matrix.

Gross and histologic evaluation of surface epithelialization correlated well; wounds were 80% to 99% epithelialized as determined by both methods. Of interest, Dermagraft-TC was fully incorporated as a neodermis in this mouse model even though it is not used in this manner in human burn patients. The length and depth of the healed wound were evaluated histologically (Fig 2). ADM produced a significantly thicker (*P* < .04) neodermis compared to the control.

**Figure 3:**
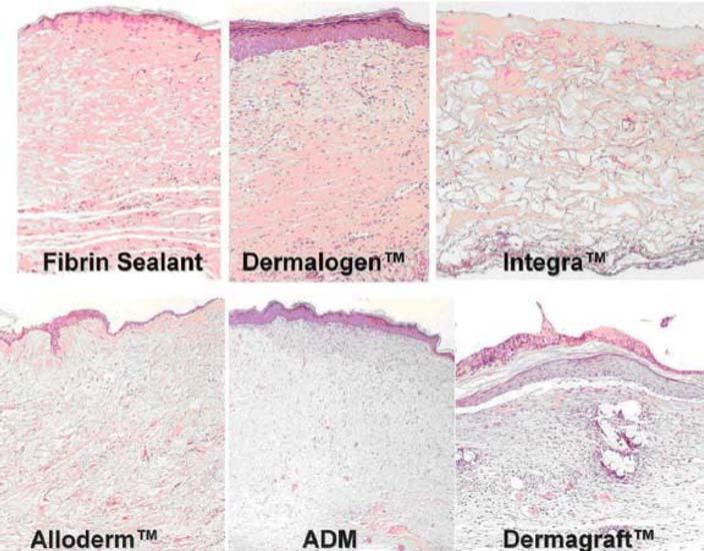
Cross sections of wounds showing epidermal regeneration over dermal substitutes. ADM indicates acellular dermal matrix.

Mouse keratinocytes migrated inward from the wound margins to regenerate the epidermis. The normal uniform 4-cell layer thick epidermis was replicated in control and Alloderm groups, but Integra, Dermagraft-TC, and Dermalogen groups exhibited a significantly thicker epidermal layer than did the control group, *P* < .04. Integra, Alloderm, and Dermagraft-TC groups showed a highly variable number of cell layers (1–5) in the epidermis (Fig 3).

### Neodermis

The healing process was characterized by mouse macrophages and fibroblasts initiating the repair and deposition of new fibrous tissue above and below the dermal substitutes enveloping them under an epidermal layer. New blood vessels and capillaries were observed within the dermal substitutes and the new fibrous tissue beneath the dermal substitutes. Histologically, the healed wound area was well delineated due to the lack of hair follicles and adnexa, which remained at and outside the healed wound margins. Because the dermal substitutes extended laterally to a greater extent than visualized grossly, the microscopically recorded healed wound lengths were increased in all groups; ADM and Alloderm groups maintained the original wound dimensions after healing, with the least contracture of the wound surface area (Table 1).

### Miscellaneous

The histologic characteristics of 28-day wounds are shown in Table 2.

The Integra matrix was encased in fibrous tissue with a significantly greater number of FBG cells compared to the control group and the other study groups, *P* < .003. Squamous hyperplasia, a reactive florid overgrowth of the epidermal layer, was seen most frequently with Integra, Dermagraft-TC, and Dermalogen. Squamous pearls (a result of keratinocyte degeneration) were prominent in Integra (83%) and control (71%) groups, followed by Alloderm (50%), Dermagraft-TC (40%), and Dermalogen (22%) groups but were absent from the ADM group (*P* < .04). By Day 28, Integra and Dermagraft-TC (dermal substitutes with the least resemblance to human dermis) were not incorporated into the fibrous dermal network and granulation tissue as well as were Alloderm, Dermalogen, and ADM. Histologically, the Integra framework and Dermagraft-TC polymers and membrane were still evident, not resorbed, although the wounds were healed. Within-group and control comparisons showed no significant differences with respect to the presence of dystrophic calcification, squamous hyperplasia, extent of granulation tissue, numbers of infiltrating neutrophils, fibroblasts, and macrophages, epidermal keratinocyte stratification, or collagen fiber configuration.

### Integra

In contrast to the other dermal substitutes used here, Integra showed the least propensity for a fibrous dermal restructuring by Day 28; squamous pearls filled the crevices of the Integra “scaffold,” and the hyalinized interstitium was extensively infiltrated by FBG cells. In the healed wound, the Integra matrix was enveloped by fibrous tissue with “islands” of Integra isolated by the ingrowth of fibers into areas that had been cleared by FBG cells. This was a morphology unlike that seen with any of the other dermal substitutes. Horizontal fibers, both superficial and deep, arrayed parallel to the tissue interfaces surrounded and infiltrated the other dermal substitutes in the healed wound areas.

### Elastin

The newly formed fibrous tissue in all groups contained few elastin fibers. Some of the dermal substitutes contained intrinsic elastin fibers in varying quantities. There were no elastin fibers seen in the control or Integra group, but fibers were prominent in wounds implanted with Dermalogen as short, stubby thick strands. They were also present to varying degrees in wounds implanted with Dermagraft-TC, Alloderm, and ADM. Slim threads that stained with the Elastin Van Gieson stain were seen in the newly formed fibrous tissue enveloping the Dermagraft-TC–implanted healed wound area. To a lesser degree, ADM and Dermalogen groups showed elastin strands that may have been displaced in the healing process into the newly generated fibrous tissue around the periphery of the implanted dermal substitute.

## DISCUSSION

### Wound contracture

All full-thickness wounds implanted with/without dermal substitutes produced epithelialized healed wounds without dermal appendages by Day 28 postsurgery. The dermal substitutes were incorporated into the healed wounds to a greater or lesser extent depending on the initial composition of the substitute. Although there was histologic variability in the composition of the healed wounds, all dermal interventions decreased wound contraction and supported the formation of an epidermis. ADM and Alloderm, both human skin–derived materials, showed less contraction in the healed wounds compared to the control or the non-human skin–derived dermal substitutes. Dermalogen, while replete with fragmented elastic fibers, remained amorphous during healing and expanded along the wound margins, resulting in a thinner healed dermis. The control, which had only Fibrin Sealant on the wound surface, also decreased wound contracture by retaining 30% of the original wound area. The use of fibrin glue as a “dermal substitute” had been previously described.^12^ Although it decreased wound contracture in this study, Fibrin Sealant was not as effective as the other dermal substitutes in creating a substantial neodermis.

### Epithelialization

All wounds implanted with dermal substitutes were epithelialized. Integra and ADM require iatrogenic intervention in patients (eg, for skin grafting), but in this model, competent mouse-derived epidermal layers (although thin) formed over the matrix without the need for additional skin grafting. Reports in the literature indicate that adult mouse bone marrow cells can differentiate into all skin components such as epidermal keratinocytes, sebaceous gland cells, and epithelial, dendritic, and endothelial cells.^13^ It is unknown at this time whether the epidermal layer expanded from the epidermis along the wound margin or formed from adult mouse bone marrow cells that populated the area. Dermalogen and Integra showed a propensity for squamous hyperplasia, an exuberant regeneration of the epidermal layer. Human burn scars may develop pseudoepitheliomatous hyperplasia (disordered progression of squamous hyperplasia), with a proclivity toward aneuploidy, which may predispose toward the development of squamous cell carcinoma.^14^ The present study showed that while squamous hyperplasia was seen, all wounds healed without evidence of malignant progression. Current literature reporting long-term follow-up of patients who received these dermal substitutes has shown no tendency toward the development of malignancy.

### Neodermis

Successful treatment with dermal skin substitutes requires low antigenicity, the capacity for rapid vascularization, and stability as a dermal template.^7^–^9^ Dermal substitutes provide stability to split-thickness skin grafts and to cultured epithelial autografts. In this study, the dermal substitutes may have formed a barrier that prevented hair follicle and skin adnexa reconstitution within the 28-day study period. The most acceptable dermal substitutes were derived from full- or split-thickness allogeneic skin treated to remove epithelial components (keratinocytes, sweat glands, and sebaceous glands) and dermal components (fibroblasts, vascular endothelium, and smooth muscle) compared to those derived from xenogenic materials (pig or bovine skin, shark cartilage, etc). “Scaffolding” in ADM and Alloderm consisted of already well-formed fibrous tissue. In contrast, Integra required a dissolution of its “foreign body scaffolding” before the more permanent fibrous tissue could be laid out in the area it occupied. The human skin–derived dermal substitutes provided a thicker and longer dermis because they had, albeit altered, greater mass to start with in terms of collagen, and other fibers into which the macrophages and fibroblasts could infiltrate, initiating angiogenesis and dermal substitute incorporation.

### Integra

It has been previously reported that 14.4% of human patients who received Integra developed the FBG cells and eosinophils.^15^ The present study showed numerous FBG cells in the Integra-implanted healed wound. This was probably a reaction to the constituents (ie, denatured bovine collagen and shark chondroitin sulfate) of Integra, which were perceived as foreign bodies. It is possible that this process contributed to the thinner “new dermis” observed here with Integra and it deserves further investigation, especially at later postsurgical intervals. This foreign body reaction to Integra components in some patients may be impacting the generation and vascularization of the new dermis as the wound heals.

### Elastin

Although it takes years for elastin fibers to become established in human scars, elastin fibers may form as early as 90 days into the wound healing process in mice.^16^ While elastin has a complex structure, there have been reports of earlier elastin deposition, as early as 40 days after grafting in humans.^16^–^19^ Dermagraft-TC when used as a dermal substitute appeared to promote the formation of elastin fibers earlier than did other dermal substitutes. Further study is needed to determine whether the elastin fibers were newly formed or whether they were transposed or redistributed from the dermal substitute during the progression of the fibrous network repair.

### Potential areas of investigations

This study, while showing that the human skin–derived dermal matrices formed the thickest neodermis and produced the least amount of contracture, was a relatively small study that investigated wound healing during a limited time period. Epithelialized wounds remodel continually for more than a year. Several questions were raised in this study that merit continued comparison of dermal substitutes and their long-term effects on wound healing. It is unknown whether the characteristics of each dermal substitute, observed at 28 days, would have been sustained for longer time intervals. Do patients receiving Integra as a dermal substitute retain the same skin pliability several years later? Why do some dermal substitutes elicit a FBG cell reaction and others do not? How effective is Fibrin Sealant alone as a dermal substitute? Do these dermal substitutes perform differently in burn/excised wounds? Investigations are underway to assess these questions, and to determine the long-term sequelae and wound remodeling characteristics of these dermal substitutes.

## Figures and Tables

**Table 1:** Dimensions of healed wounds treated with dermal substitutes at 28 days postsurgery: gross and histologic evaluations

**Parameters**	**Control**	**Integra**	**Alloderm**	**Acellular dermal matrix**	**Dermagraft-TC**	**Dermalogen**
# Cases	7	6	6	6	5	6
% Total surface wound*	34 ± 7†	46 ± 12	63 ± 14†	57 ± 7†	50 ± 22	39 ± 9
% Surface epithelialization*	100 ± 1	92 ± 7	86 ± 9	87 ± 10	82 ± 10	95 ± 8
Surface wound length, mm‡	5 ± 2	7 ± 2	9 ± 6	10 ± 2	8 ± 3	7 ± 2
Under surface wound length, mm‡,§	6 ± 2	8 ± 2	12 ± 3	12 ± 1	9 ± 3	9 ± 3

Neodermis depth, mm‡	0.7 ± 0.1	1.3 ± 0.6	0.95 ± 0.5	1.4 ± 0.3	0.8 ± 0.24	1.0 ± 0.3

*Gross measurement based on a 4 cm^2^ wound area.

^†^*P* < .03 compared to control.

^‡^Histologic microscopic measurement.

^§^Two-dimensional length beneath the epidermal layer without the presence of hair follicles and adnexa.

**Table 2:** Histologic characteristics of the 28-day postsurgery healed wound area*,†

**Parameters**	**Control**	**Integra**	**Alloderm**	**Acellular dermal matrix**	**Dermagraft-TC**	**Dermalogen**
# Cases	7	6	6	6	5	6
% Squamous hyperplasia	14	67	33	17	40	67
% Squamous pearls	71	83	50	0	40	17
% FBG cells	0	100	0	33	0	17
% Dystrophic calcification	0	17	0	0	20	17

*Percentages reflect the frequency of histologic features within each study group.

^†^FBG indicates foreign body giant.
